# An Investigation of the Dynamical Transitions in Harmonically Driven Random Networks of Firing-Rate Neurons

**DOI:** 10.1007/s12559-017-9464-6

**Published:** 2017-04-07

**Authors:** Kyriacos Nikiforou, Pedro A. M. Mediano, Murray Shanahan

**Affiliations:** Department of Computing, Imperial College London, London, UK

**Keywords:** Attractor dimensionality, Recurrent neural network dynamics, Activity visualization, Driven networks, Dimensionality embedding

## Abstract

Continuous-time recurrent neural networks are widely used as models of neural dynamics and also have applications in machine learning. But their dynamics are not yet well understood, especially when they are driven by external stimuli. In this article, we study the response of stable and unstable networks to different harmonically oscillating stimuli by varying a parameter *ρ*, the ratio between the timescale of the network and the stimulus, and use the dimensionality of the network’s attractor as an estimate of the complexity of this response. Additionally, we propose a novel technique for exploring the stationary points and locally linear dynamics of these networks in order to understand the origin of input-dependent dynamical transitions. Attractors in both stable and unstable networks show a peak in dimensionality for intermediate values of *ρ*, with the latter consistently showing a higher dimensionality than the former, which exhibit a resonance-like phenomenon. We explain changes in the dimensionality of a network’s dynamics in terms of changes in the underlying structure of its vector field by analysing stationary points. Furthermore, we uncover the coexistence of underlying attractors with various geometric forms in unstable networks. As *ρ* is increased, our visualisation technique shows the network passing through a series of phase transitions with its trajectory taking on a sequence of qualitatively distinct figure-of-eight, cylinder, and spiral shapes. These findings bring us one step closer to a comprehensive theory of this important class of neural networks by revealing the subtle structure of their dynamics under different conditions.

## Introduction

Continuous-time recurrent neural networks are prevalent in multiple areas of neural and cognitive computation. They have been successfully used as models of cortical dynamics and function [1, 2] and have also found application in machine learning [3–8]. In biological modelling, it is important to know how networks respond to external forces, as neural circuits are constantly receiving stimuli from the environment and other brain regions [9, 10]. In a machine learning context, it is important to know how external inputs affect the behaviour and expressive power of the model. Furthermore, it has long been proven that these networks can approximate any dynamical system to arbitrary precision [11, 12], but further empirical study is needed to understand the practicalities of such approximations and how network dynamics are shaped by incoming stimuli [13]. A characteristic phenomenon exhibited by such networks is a qualitative change in their dynamics—depending on the precise values of some of the parameters—commonly referred to as a bifurcation. This phenomenon has been studied analytically for the case of networks with less than ten neurons [14, 15] but to take an analytical approach to larger networks comprising hundreds of neurons would be very challenging. Hence, numerical approaches are the main tool for investigating how these bifurcations appear and what their effect is on network dynamics.

The goal of the present article is to investigate, both visually and numerically, the properties of externally driven recurrent neural networks. We build on previous work by Sussillo and Barak [16] and focus in particular on two key aspects of network dynamics: temporal evolution of stationary points and attractor dimensionality. We extend established visualisation techniques to illustrate and understand the relation between these underlying dynamical properties and actual instantiations of the network’s trajectory through its state-space. Finally, we uncover the coexistence of underlying attractors with various geometric forms but the same dimensionality in unstable networks.

This paper is organised as follows. In “Methods”, we present a novel approach to stationary point dynamics and introduce a technique to measure attractor dimensionality adapted from Tajima et al. [17]. In “Results”, we show the results of our analysis on various networks, and focus on the differences between stable and unstable regimes. Specifically, we consider timescales of the network’s dynamics that are relevant to that of the input signal. What we mean by this is that the timescale at which we examine the complexity of the network’s response is comparable to the timescale at which the harmonic input oscillates. In terms of the network’s information processing capabilities, this timescale is the most relevant one. In other words, networks that show rich, complex dynamics at this timescale can theoretically perform the most interesting nonlinear transformations of the input [5], from a computational point of view. Finally, “Discussion” evaluates the methods used and discusses the significance of attractor dimensionality for reservoir computing applications and biological modelling.

## Methods

### Network Simulations

#### Neuron Model

The neuron model used in these experiments was the continuous-time firing-rate model [18, 19]:

1a $$\begin{array}{@{}rcl@{}} &&{} \tau \frac{dx_{i}}{\text{dt}} = -x_{i} + \sum\limits_{j=1}^{N} W_{\text{ij}}^{\text{Res}} r_{j} + w_{i}^{In}S(t) \end{array} $$

1b $$\begin{array}{@{}rcl@{}} &&{}r_{i} =\tanh(x_{i}) , \end{array} $$

where *x*_*i*_ is the membrane potential of neuron *i*, *τ* is a time constant that determines the timescale of the neuron’s activity, $W_{\text {ij}}^{\text {Res}}$ is the connection weight from neuron *j* to neuron *i* and $w_{i}^{In}$ is the connection weight from the external input *S* to neuron *i*. We considered networks driven by an external oscillator $S(t) = \sin (\alpha t)$, and studied the response of the network for varying frequencies *α*. All neurons received input from the external oscillator with a weight $w_{i}^{In} \sim \mathcal {N}(0, 1)$. The recurrent connections *W* ^Res^ were given by a random Erdös-Renyi graph with connection probability *p* = 0.1, no self-connections and weights $W_{\text {ij}}^{\text {Res}} \sim \mathcal {N}(0, {g_{G}^{2}})$, with $g_{G} = g/\sqrt {pN}$ acting as a global scaling factor. We refer to *g* as the *gain*, an important parameter that strongly affects the dynamic behaviour of the system. It has long been established [19] that autonomous networks with *g* < 1 exhibit attracting dynamics towards a globally stable stationary point, whereas networks with $g\geqslant 1$ can exhibit complex periodic or even chaotic behaviour. On the other hand, the activity of input driven networks is heavily dependent on the input signal. In some cases, this input has even been shown to suppress chaotic activity for values of *g* greater than 1 [13], indicating the impact of the input signal on the network’s activity. For convenience, we refer to networks with *g* < 1 as stable networks and those with $g\geqslant 1$ as unstable. Finally, the variable *r*_*i*_ in Eq. 1b is the observed activation, or firing rate, of neuron *i* and lies in the interval [−1,1].

Notice that with this sinusoidal input, the only quantity determining the network’s response and complexity is the relative timescale of the neurons and the stimulus. Therefore, for ease of analysis and without loss of generality we can reparametrise $\hat {t} = \tau ^{-1} t$ in Eq. 1a as

2 $$ \frac{dx_{i}}{d\hat{t}} = -x_{i} + \sum \limits_{j=1}^{N} W_{\text{ij}}^{\text{Res}} r_{j} + w_{i}^{In}\sin \left( \rho \hat{t} \right) ,  $$

where the only parameter of interest is now the ratio between the neuron timescale *τ* and the period of the driving force^1^ *τ*_*F*_, i.e.

3 $$ \rho = 2\pi\frac{\tau}{\tau_{F}} .  $$

#### Numerical Simulations

In order to change the value of *ρ* between different simulations, we fixed the frequency of the sinusoidal input at $\frac {10}{2\pi }$, such that $\tau _{F} = \frac {2\pi }{10}$, and instead varied *τ*. This way, the resolution of the sinusoidal input was the same for all simulations. We note here that increasing *τ* is mathematically equivalent to increasing the frequency of the oscillation, as shown from Eqs. 2 and 3. Simulations were performed using the Euler integration method with step size 0.01 and were run for 3500 timesteps. The networks were initialised randomly and received a short, strong pulse of amplitude 5 through *w* ^*In*^ after 200 timesteps, for 50 timesteps. The purpose of this pulse was to guide the network to a bounded region of its state-space with a small hyper-volume. The oscillatory input was then applied. After discarding the first 1500 points of the simulations as transients, the network activity for the remaining simulation was analysed. A different set of input and network connectivity weights, *w* ^*In*^ and *W* ^Res^, respectively, were generated for each simulation. Finally, for finding the candidate points of Eq. 5, the MATLAB function fsolve was used with the trust-region-dogleg algorithm [20].

### Analysis and Visualisation Techniques

#### Stationary Point Analysis

Loosely speaking, a stationary (or fixed) point in a dynamical system’s state-space is one where it will remain if initialised there and not subject to external input or noise. More formally, it is a point where all components of the system’s gradient are zero. Stationary points can be classified as sources, sinks, limit cycles or saddles for low-dimensional systems, and can get more complicated as the dimensionality of the state-space increases. Stationary points are key to predicting the long-term behaviour of the system and its response to perturbations. Here we extend the work of Sussillo and Barak on stationary point analysis in recurrent neural networks [16], and refer the interested reader to [21, 22] for a review of stationary point analysis techniques in nonlinear dynamical systems.

For finding the stationary points of the driven reservoir, the system of equations describing the evolution of the network can be rewritten in matrix form for a constant stimulus, as

4 $$ F({x}, s) \triangleq {\dot{{x}}} = \frac{1}{\tau} \left( -{x} + {W}^{\text{Res}}{r} + {w}^{In}s \right) .  $$

Formulated this way, we see that finding the stationary point *ϕ* of the system for a given input current *s* is equivalent to solving the fixed-point vector equation *F*(*x*,*s*) = 0. In other words,

5 $$ \phi(s) = \arg_{\text{x}}\min \left|F({x}, s)\right|^{2} .  $$

The most common problem in finding stationary points is the practical feasibility of this optimisation—in the standard reservoir computing scenario the number of neurons in the network ranges from several hundred to several thousand, making full optimisation intractable. We overcome this problem by leveraging the smoothness of the network’s dynamics to find stationary points for constant non-zero values of *s*, and formulating our approach as a set of sequential optimisation problems. Starting from the trivial solution *ϕ*(0) = 0, the value of *s* is gradually incremented in small steps Δ, calculating *ϕ*(*s*) at each step. Importantly, this allows us to use the result of the previous step *ϕ*(*s*) as the starting point for the next optimisation for *ϕ*(*s* + Δ), facilitating convergence of the optimisation and making the whole method tractable and effective. To ensure consistency in our experiments, an optimisation was only considered successful if all elements of the gradient vector had an absolute value below 10^−15^. In our simulations, we found that incrementing *s* in steps of Δ = 0.01 offers a good balance between convergence probability and speed. Once *ϕ*(*s*) is successfully computed for all relevant values of *s* (in our case, a grid in the [−1,1] interval), the temporal trajectory of the stationary point can be easily described by *ϕ*(*S*(*t*)).

#### Locally Linear Dynamics

In order to characterise the local dynamics of the network near the stationary points, we can approximate Eq. 1a by a 1st-order Taylor expansion. We can then analyse the Jacobian of the linearised system, which following Eq. 4 is defined as

6 $$ {J} = {W}^{\text{Res}}\circledast{B} - {I} ,  $$

where $\circledast $ denotes an element wise multiplication between the two matrices, *B* is an *n* × *n* matrix with identical rows $B_{\text {ij}} = (1-\tanh (x_{j})^{2})$ and *I* is the identity matrix.

The Jacobian was calculated at each stationary point, and then diagonalised. This allowed us to extract the eigenvectors corresponding to the eigenvalues of the linearised system. In order to determine the nature of the stationary point, we focused on the two largest eigenvalues of the linearised system and used results from dynamical systems theory to determine the nature of the fixed point. For the cases in which the second largest eigenvalue was a complex number, we also considered the third largest eigenvalue, which is its complex conjugate, and extended results for two-dimensional systems to three dimensions by considering linear dynamics along a plane and a vector, as opposed to only two vectors [22]. It is known that if the two largest eigenvalues are real and negative, the stationary point behaves as a sink along the plane spanned by the two eigenvectors; if these eigenvalues are both positive, it acts as a source; and if they are mixed as a saddle point.

The challenge for visualisation comes if the eigenvectors have a non-zero imaginary part, as is often the case. Dynamically, this indicates the presence of rotational dynamics near the stationary points. To visualise this effect, the diagonal form of the Jacobian at each stationary point can be transformed into its real Jordan canonical form, where pairs of complex conjugate eigenvectors are transformed into a pair of real vectors that define a basis for the hyperplane that the rotation lies on. This pair of real eigenvectors directly determines the orientation of the rotation plane of the locally linear dynamics.

#### Dimensionality of Attractor Dynamics

While the stationary points and their local dynamics provide a great deal of information about the response of the network to constant input, all experiments presented in “Results” were run with time-varying stimuli. An interesting global property of network dynamics is the fraction of all possible states that the network actually visits, as this affects the richness of its dynamical repertoire and consequently its expressiveness. It is possible, and in fact common, for the network to have a finite *attractor dimensionality* *D*, such that the dynamics of the network effectively lie on a manifold of dimension *D*, which is much smaller than the number of neurons *n*. A higher attractor dimensionality indicates more complex dynamics, which in turn allow the reservoir to engage in more sophisticated computation. In this article, we explore and compare two techniques for estimating attractor dimensionality, one based on linear methods and one based on more general, non-parametric estimators.

The linear estimator *D*_PCA_ is based on the principal component analysis (PCA) of the network’s trajectory. The attractor dimensionality is then simply defined as the minimum number of principal components (PC) required to explain 95% of the variance in the network’s trajectory. The measure was averaged over multiple runs of the network for the results presented in this study.

The calculation of the nonlinear, non-parametric estimator *D*_kNN_ is slightly more involved. First, we select two time series of the activation of same neuron in different runs of the simulation and calculate the delay-embedding vector of one of them [23]. Then standard *k*-nearest neighbour regression [24] is used to estimate the next value of one time series based on the embedding vector of the other, using four nearest neighbours and a delayed-embedding step of *τ*_*d*_ = 4. Analogously to *D*_PCA_, *D*_kNN_ is defined as the minimum dimensionality of the embedding vector such that the regressed values capture more than 95% of the variance of the trajectory. This estimation was also averaged over multiple neurons and multiple runs of the same network. For details on this method we refer to the original article by Tajima et al. [17]. We finally note that ideally one would like to explain 100% of the variance in order to identify the dimensionality of the attractor, but here we allow for some flexibility and consider 95% as a reasonable compromise, which is in agreement with Tajima et al. [17].

## Results

### Stable Networks

#### Stationary Point Trajectory

**Fig. 1:**
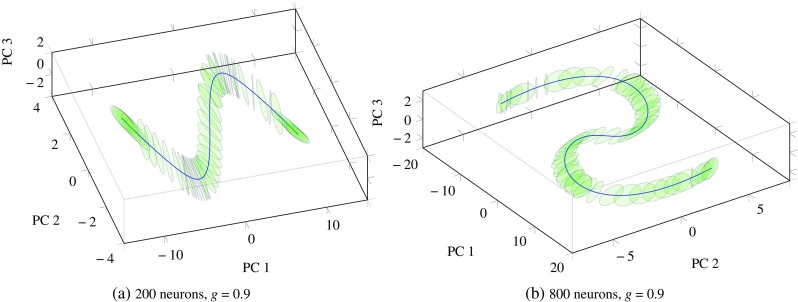
(*blue*) Trajectories of the stationary points visited as the input varies sinusoidally, projected onto their three PCs. The origin represents the stationary point for the case where the input is zero, and the two extrema of the curves represent the case where the inputs are −1 and 1, respectively. (*green*) Attracting plane spanned by the pair of complex conjugate eigenvectors corresponding to the pair of eigenvalues with the highest absolute value at each stationary point

First, we applied the stationary point analysis technique described in “Analysis and Visualisation Techniques” to two networks of different sizes, both slightly under the edge of chaos with *g* = 0.9. We performed PCA on the stationary point trajectories and plotted a projection of these trajectories on their three first PCs in Fig. 1. For all stationary points and both networks, the eigenvalues with the largest absolute value—which influence the dynamics near the point most strongly—were complex conjugate pairs with negative real parts. This indicates that the local dynamics exhibit an attracting rotational effect towards the stationary points—i.e. if injected a constant input, the network “spirals down” to the stationary point along the green circles in Fig. 1.

The first point of interest in Fig. 1 is the regularity with which the stationary point shifts as the input changes, resulting in a smooth trajectory in the reduced PC-space. This validates the rationale behind the sequential optimisation approach which allowed the optimisation to be completed much faster than by using a random initial point. The second point of interest is that the planes formed by the two most dominant eigenvectors also change smoothly along the stationary point trajectory. This can be linked to the fact that even in the presence of a varying input of the form $w_{i}^{In}S(t)$, the network remains in the stable dynamical regime and undergoes a smooth deformation of its locally linear dynamics near the stationary points. We note that this method is applicable to larger networks and we observe that the stationary point and its local dynamics also change smoothly with varying input (results not shown).

For each value of the input *s*, we can interpret the behaviour of the network as following a vector field *F*(*x*,*s*) which, if given enough time, will bring the network to *ϕ*(*s*). However, in our simulations the input was changing with time. This means that the network did not completely follow the trajectory of the fixed point, but operated under the action of a different vector field at every timestep. The parameter *ρ*, being the ratio between the timescales of the neurons and the input, regulates how fast the stationary points move back and forth along their trajectory and how fast the network moves in its state-space.

**Fig. 2:**
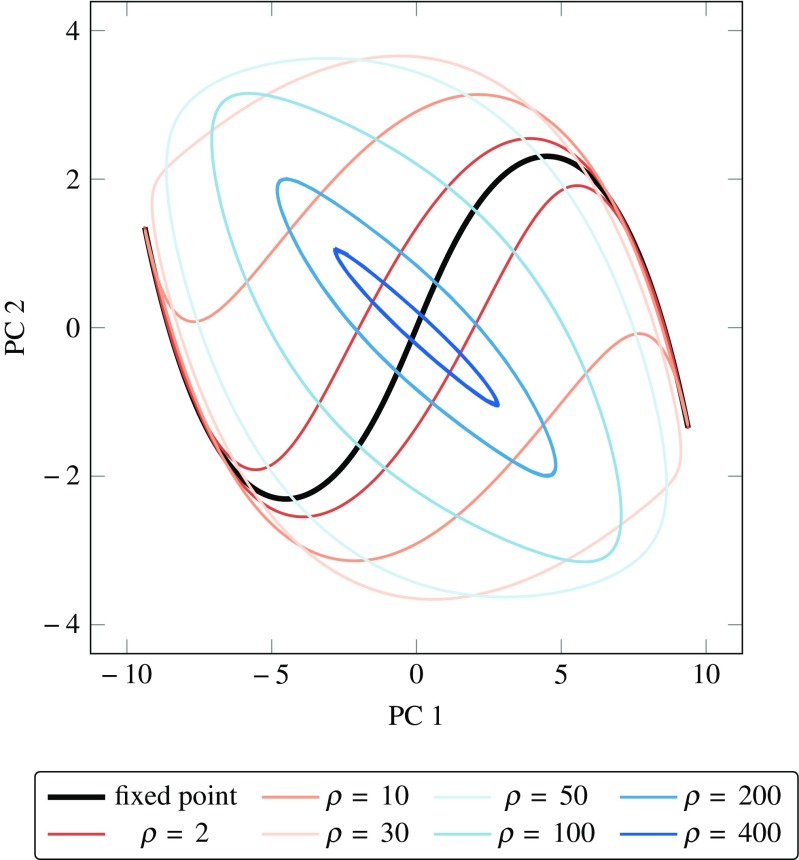
Set of stationary points (*centre black curve*) and trajectories of an *N* = 200 stable network for different *ρ* values. All trajectories have been projected onto the two largest principal components of the set of points visited by the stationary point. Projected network trajectories for lower *ρ* values lie closer to the projected stationary points, indicating that the fixed points move at a *speed* that is comparable to that of the network’s evolution and can hence *pull* the network activity close to them. As *ρ* increases to about 30, the projected network activity initially opens up and then, for $\rho \geqslant 50$, falls into an oscillation mode that is orthogonal to the oscillation mode of the stationary points in reduced PC-space, while condensing around the origin. This illustrates that as *τ* becomes much larger than *τ*_*F*_, the network activity progressively moves into a subspace that is orthogonal to the two principal components of the stationary point trajectory, indicating a *disentanglement* of the two trajectories

**Fig. 3:**
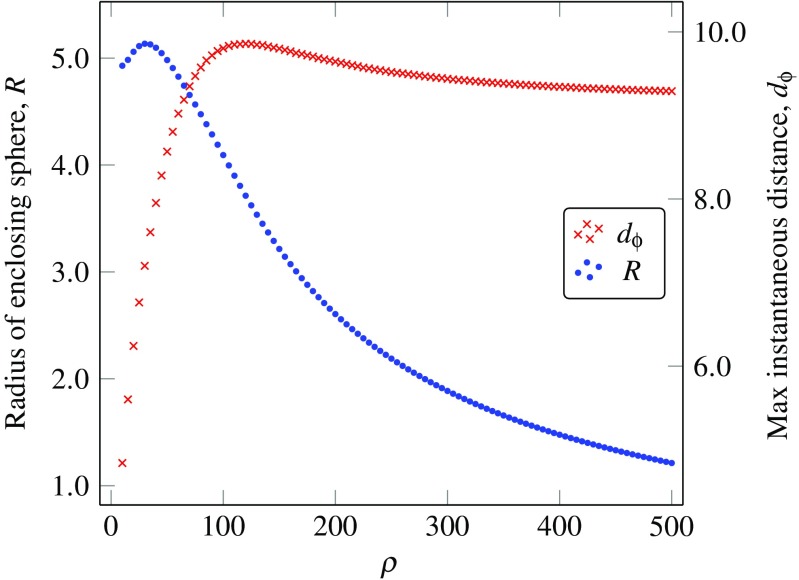
Stable network with *N* = 200 neurons and *g* = 0.9. (*blue*) Radius of the smallest enclosing sphere in the reduced PC-space. (*red*) Maximum instantaneous distance in the reduced PC-space between the network state and the stationary point trajectory

To visualise this interplay between a changing vector field and the network’s state, we plotted the activity of the same network driven by signals of different *ρ* values, projected and overlaid in the space of the set of stationary points (Fig. 2). For each trajectory, we calculated the radius *R* of the smallest enclosing sphere in the reduced space and its maximum instantaneous distance *d*_*ϕ*_ from the stationary point (Fig. 3). These results match our intuitions—for lower values of *ρ*, the stationary point moves slowly along its trajectory and at a speed through the state-space which is comparable to the speed at which the network activity evolves. Due to the fact that all stationary points remain sinks, the state of the network is being constantly pulled by the moving stationary point and remains close to it, yielding a small *d*_*ϕ*_. As *ρ* increases, the stationary point moves faster and can no longer be followed by the network state, such that the trajectories of the network become wider and progressively less similar to that of the stationary point, as reflected by the rapidly increasing *d*_*ϕ*_. Finally, as *ρ* gets much larger, the vector field changes much faster than the network can accommodate and trajectories start condensing around the origin in the reduced PC-space. Eventually *R* vanishes into a subspace orthogonal to the reduced PC-space and *d*_*ϕ*_ asymptotically approaches the maximum distance of the set of stationary points to the origin. This happens because the network activity is no longer able to follow the moving stationary point closely, due to the fact that the stationary point and network state move with very different velocities.

This behaviour is loosely reminiscent of the resonance behaviour observed in conventional driven harmonic oscillators. If the oscillation of the driving force is slow compared to the intrinsic timescale of the oscillator, the system follows the trajectory imposed by the force. With higher frequencies of the driving force the amplitude of the resulting oscillation increases, as resonance effects start playing a role. Finally, for even faster frequencies, the force changes too fast for the system to assimilate, and the amplitude of the oscillation shrinks around the origin.

#### Attractor Dimensionality

**Fig. 4:**
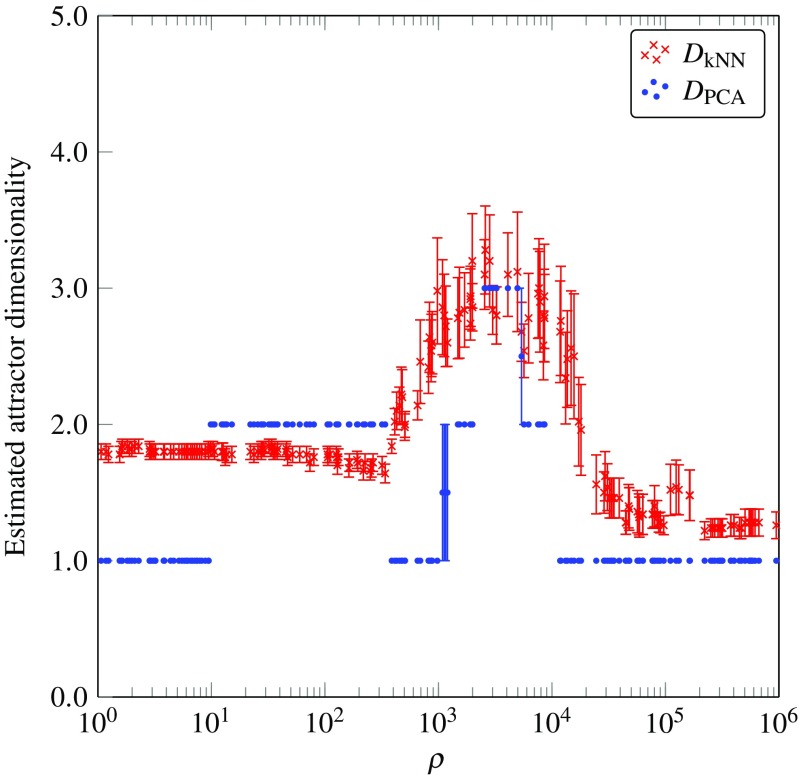
Underlying attractor dimensionality of stable networks with *N* = 200 neurons and *g* = 0.9 for different *ρ* values, with the two proposed estimators. *Error bars* denote the standard error of the estimated dimensionality from different neurons and runs of the same network

**Fig. 5:**
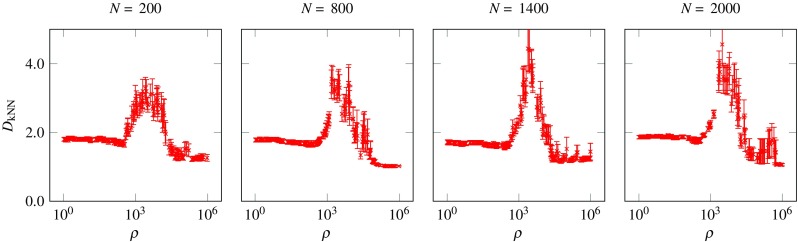
Estimated underlying dimensionality *D*_kNN_ for stable (*g* = 0.9) networks of different size and different *ρ* values. A different network with random connectivity was created for each data point. *Error bars* denote the standard error of the estimated dimensionality from different neurons and different runs of the networks

Next, we studied the effect of the driving frequency on the dimensionality of the attractor, as measured by both *D*_PCA_ and *D*_kNN_. The discussion focuses on the estimates obtained with *D*_kNN_, which are expected to be more reliable. The main reason for this is that the nonlinear estimator is not affected by the different scales of the independent dimensions of the attractor while the linear estimator is very sensitive to them. Estimates for *D*_PCA_ are also presented for comparison and to illustrate the limitations of applying linear assumptions for measuring the dimensionality of nonlinear attractors. Figure 4 shows the estimated dimensionality of a stable *g* = 0.9 network with *N* = 200 neurons, driven by external oscillators of varying *ρ*. The first insight we obtain is that the driving frequency can radically change the dimensionality of the network’s activity, ranging from as low as 1 to as high as 4. For low values of *ρ*, the attractor dimensionality was about 2, and remained approximately constant until *ρ* ≈ 400. As we increased *ρ*, the dimensionality started increasing, showing a strong and clear peak around *ρ* ≈ 3000, and for very high *ρ* fell back to 1. The trends in dimensionality for very high values of *ρ* are discussed in detail in “Very High Values of *ρ*”. We also note the low variance of *D*_kNN_ across trials of a given frequency, in particular for very low and very high *ρ*. Furthermore, we explored how these results scale for networks of different sizes (Fig. 5). Larger networks show very similar behaviour, with a peak in complexity for intermediate values of *ρ*. Finally we note that a clear discontinuity in networks with *N* = 800 and *N* = 2000 was observed during the increase in dimensionality, that could potentially indicate a sharp phase change. A similar discontinuity was not observed for networks with *N* = 200 or *N* = 1400. Additional simulations were carried out to examine this transition near the peak (results not shown), using different variance thresholds and different *k*-NN regression schemes. This investigation showed that while the trend of dimensionality change shown in Fig. 5 is robust for various variance thresholds and regression schemes (with some noise in the estimated value), the appearance of discontinuities is sensitive to the variance threshold.

### Unstable Networks

The results presented in the “Stable Networks” correspond to stable networks, which exhibit stable dynamics in the presence of constant input, due to the presence of sink attractors. As has been shown in the previous section, these networks can still produce interesting behaviour if driven by a time-varying external signal. On the other hand, autonomous networks with a higher gain, *g* > 1, are regarded as unstable in the sense that their activity remains complex without converging to a stable state, due to the presence of multiple saddles in their vector fields [16]. For the case of externally driven networks with *g* > 1, previous work [13] has shown that a strong oscillatory input can *entrench* the network dynamics and induce periodic, nonchaotic network activity. The focus of this study is to visualise the dynamical *skeleton*, i.e. the position and nature of stationary points, with the aim of understanding how the instantaneous value of the input changes the vector field that determines the dynamics of the network.

**Fig. 6:**
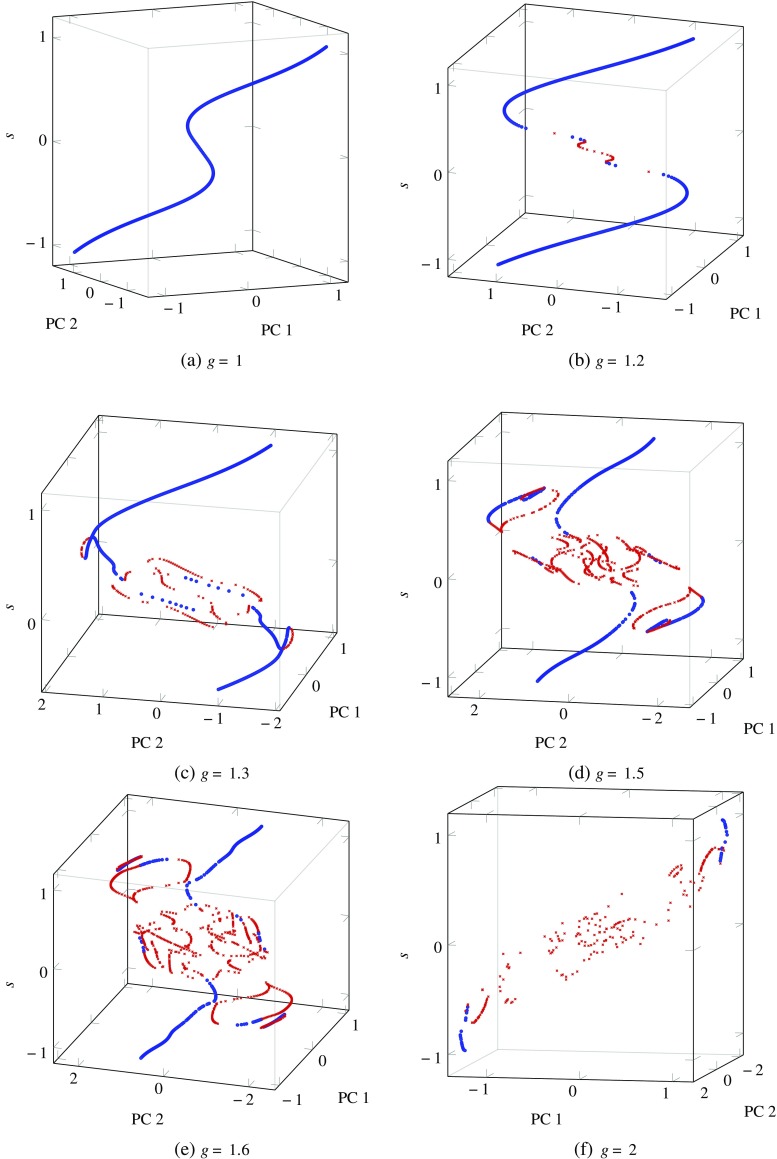
Stationary point visualisations for different values of *g* in networks of 200 neurons. Stationary points were discovered with 50 random initialisations of the optimisation procedure (similar to Sussillo and Barak [16]) for each value of the input signal *s* and only points satisfying the stationarity condition were kept. After performing an eigen-decomposition on the linearised dynamics, each stationary point was classified as a sink (*blue dots*) or a saddle (*red crosses*), depending on the sign of the real components of the eigenvalues

**Fig. 7:**
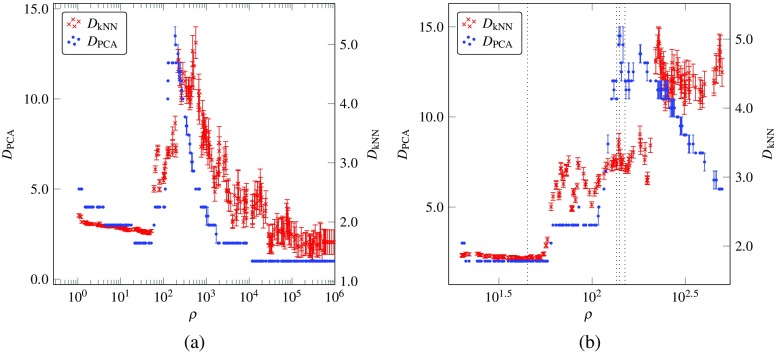
Underlying attractor dimensionality estimated by *D*_PCA_ (*blue*) and *D*_kNN_ (*red*). Estimates are presented **a** across the full range of *ρ* values and **b** for *ρ* values close to the transitions presented in Fig. 8. The *dotted lines* in **b** correspond to *ρ* values of 45, 135, 140 and 150. Simulations were carried out using an unstable network of 800 neurons and *g* = 1.5

**Fig. 8:**
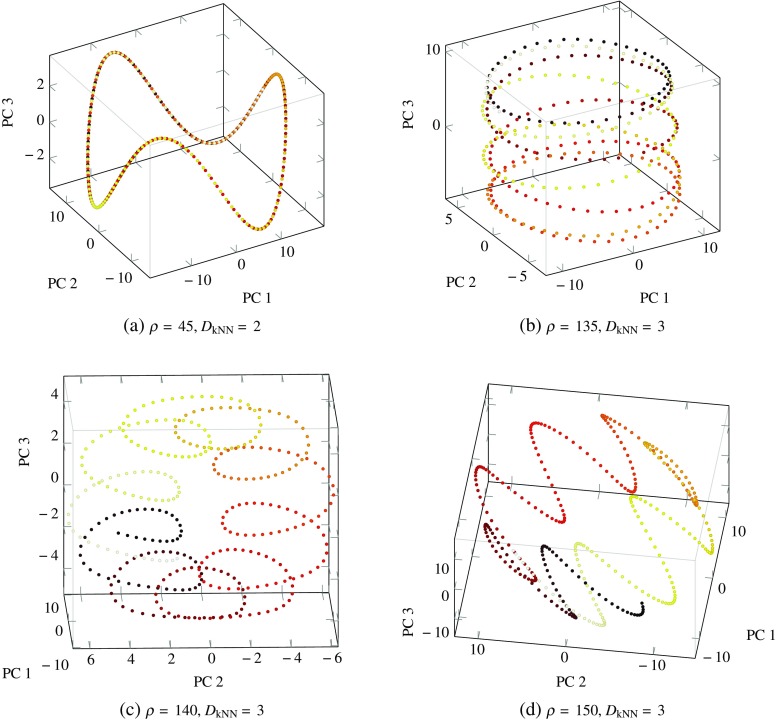
Plots of the activity for a network of 800 neurons and *g* = 1.5 driven by various *ρ* values, projected on their three principal components. The increase in attractor dimensionality that results from increasing *ρ* from 45 to 135 is associated with a transition from dynamics resembling **a** a figure-of-eight to **b** oscillations along the surface of a cylinder in the reduced space. Figures **b**, **c,** **d** show the various geometric forms of the system’s attractors with *D*_kNN_ = 3

In this section, we present results from our investigation of how the trajectory and nature of stationary points is affected by a varying input signal for networks with *N* = 200 and *g* ≥ 1 (Fig. 6). We further explore the impact of *ρ* on the activity of networks with *N* = 800 neurons and *g* = 1.5. We show how the estimated attractor dimensionality changes for different *ρ* values in Fig. 7 and illustrate their effect on the geometric form of the attractors by visualising the projections of the network’s activity for four *ρ* values in Fig. 8. We use these Figs. 6, 7 and 8 to ground our discussion and interpret dynamical transitions in the network.

#### Stationary Point Trajectory

In order to examine how the instantaneous value of the input *s* affects the nature and position of the stationary point in reduced PC-space, for networks with different values of *g*, the Sussillo and Barak [16] variant of the optimisation scheme described in “Methods” was applied. Specifically, for each instantaneous value of *s* in [−1,1], 50 random initialisations were performed for the optimisation in order to find as many stationary points as possible, of which only those satisfying the stationarity condition were kept. The eigenvalues of the linearised system at each point were examined to classify the point as a stable node or a saddle. Plots in Fig. 6 show how the position and nature of the stationary points change with *s* for networks of *N* = 200 and different *g* values. These plots convey the interesting point that both the network gain *g* and the instantaneous value of the input *s* can be considered to be bifurcation parameters, in the sense that the position and nature of the stationary points depend on their precise values. For the case of *g* < 1, this has long been established, but Fig. 6 shows that the value of *s* also plays an important role along with *g*, since the networks can remain stable even for $g \geqslant 1$, if the value of *s* is also high. More specifically, Fig. 6a for *g* = 1 shows that all stationary points remain stable nodes for all values of *s*, similar to the case of networks with *g* < 1 in Fig. 1. As *g* increases by a small amount, the range of *s* values in the vicinity of the origin generate multiple saddle points that prevent the network from reaching a stable state if run with very small or no input. This is in agreement with the well-known fact that autonomous networks are unstable for *g* > 1. As the value of *g* increases to 1.3, 1.5, 1.6 and finally 2, three trends were observed: first, the number of saddle points increases and they spread out in the projected PC-space, second, the range of *s* values where saddle points appear increases towards higher absolute values, thus increasing the fraction of the range of *s* that is unstable, and third, the number of positive eigenvalues in the saddle points increases both with *g* and with a diminishing signal amplitude. The first point was also proven from a theoretical perspective [25]; that is, the topological complexity of the state-space of firing-rate neural networks increases at a similar rate as the dynamical complexity of the network; this means that as *g* increases above 1, the number of stationary points also increases.

In light of this investigation and in agreement with previous work [26], the varying input signal can be considered as an *on-line* bifurcation parameter that can regulate the stability of the system. As the input changes from strong and positive (and thus from an operating regime with stable points) to values close to zero, a series of bifurcations occur that nudge the dynamical landscape into an unstable operating regime. This rhythmic switching between stable and unstable operating regimes results in a higher maximum attractor dimensionality for networks with *g* > 1 as opposed to networks with *g* < 1, that always experience stable nodes. We also note that the edge case *g* = 1, even though considered as an unstable case, consistently behaved similar to the stable cases (Fig. 6a).

#### Attractor Dimensionality and Geometry

Figures 7 and 8a show that for low values of *ρ* < 60, the network activity lay on a two-dimensional periodic attractor, as expected, since the network was driven by a slow sinusoidal signal with the same dimensionality. This phenomenon can be related to results from Rajan et al. [13], who showed that external stimuli can suppress chaotic activity in an otherwise chaotic network and impose a periodic behaviour on its dynamics. As the value of *ρ* was increased, the dimensionality of the underlying attractor also increased, as measured by the estimators. It is evident that the linear estimator significantly overestimated the dimensionality of dynamics compared to the nonlinear estimator and the two estimates diverged significantly in the region of *ρ* values near the peak. This effect can be attributed to a rapidly developing and highly nonlinear attractor in this region. Although the numerical values of the dimensionality differ significantly between the two estimators, the general trend for the change in dimensionality is very similar for both. Moreover, the increase in dimensionality for unstable networks resulted in a maximum dimensionality of 5 at *ρ* values that were an order of magnitude lower than the ones for stable networks, which reached a maximum dimension of 4. The higher maximum dimensionality reached in networks with *g* = 1.5 can be attributed to the spawning of saddle points in the state-space for intermediate values of the input signal. The periodic bifurcations resulting from the oscillatory signal continuously deform the state space in a non-smooth manner, as opposed to networks with *g* < 1 which experience a smooth change in the position of the stable stationary point in the state-space. As a result, the vector field experienced by unstable networks varies more with time compared to that experienced by stable networks.

Figure 8a, c, d captures the projections in reduced PC-space of attractors with a dimensionality of 3, as measured by the nonlinear estimator. It is interesting to note that the increase in dimensionality from a value of 2 to 3 for *ρ* = 45 to *ρ* = 135 can be visually observed in Fig. 8a, b. In this transition, the network activity extended from a 2-day-period attractor to oscillations around a 3D manifold, whose geometric form resembles that of a cylinder. Furthermore, in the region of *ρ* values between 130 to 150, all attractors were found to have an estimated dimensionality of about 3, but exhibited different geometric forms. Three distinct forms were found in this region and are shown in Fig. 8b, c and d. The major point of interest here is that, in addition to an increase in dimensionality as *ρ* increased, transitions between attractors of different geometric forms but the same dimensionality were observed for small changes in *ρ*. These transitions are not necessarily expected but can be explained in an intuitive manner by the insights gained in this study. Specifically, small changes in *ρ* result in the vector field changing with a slightly different frequency, while the speed with which the network evolves remains constant. Due to the appearance and disappearance of stable and saddle points as the vector field changes, the precise vector field experienced by the network as it evolves is significantly different for slightly different input signal frequencies. This results in the network orbiting around attractors that have geometrically distinct forms, as shown by the plots in Fig. 8.

Finally, we note that what can actually be visualised are not the complete high-dimensional attractors themselves, but their projections in the reduced, three-dimensional, PC-space. For this reason, care should be taken when forming interpretations from such visualisations and especially for *ρ* values that result in a dimensionality higher than 3. Nevertheless, all attractors presented in Fig. 8 had a maximum estimated dimensionality of three, as calculated with the more reliable non-parametric estimator and shown in Fig. 7. Moreover, even though the estimated dimensionality with the linear estimator was more than ten for Fig. 8b, c, d, the total variance explained by the first three principal components using the PCA estimator was about 75–80% for these plots and each subsequent dimension added a progressively smaller percentage to the total variance explained. This means that network oscillations along the plotted dimensions capture the majority of the variance of the system and any single additional dimension should not provide any additional information about the geometric form of the attractor. Hence, we conclude that enough information was retained after dimensionality reduction with PCA, in the visual sense, so as for our visual exploration to remain relevant and informative of the effect of the driving signal frequency on the dimensionality and geometric form of these attractors.

### Very High Values of *ρ*

In “Introduction”, we briefly mentioned that the focus of this study is the computational capability of networks at the timescale of the input signal. Ideally, the network should be able to perform both timely and useful output mappings when stimulated by the input signal. Networks that are not responsive to changes in the input as they occur (or shortly after), cannot be considered as able to perform useful computations on the input. In the experiments presented in this study, we varied the value of *τ*, and hence *ρ*, to examine how networks respond to input signals that operate at timescales different than those of the network. Networks that respond with higher-dimensional dynamics are more responsive to changes in the input, and hence more useful for computation, than networks that respond with lower dimensional dynamics and are hence not affected much by the changing input.

The presented results show a consistent decrease in the dimensionality of dynamics for very high values of *ρ* > 10^4^. This can be explained by the way in which the value of *ρ* was varied between different simulations. As described in “Network Simulations”, to change the value of *ρ*, the frequency of the sinusoidal input was kept constant and instead, the network’s timescale, *τ*, was changed. Consequently, for very high values of *ρ*, *τ* is also very high, due to the direct proportionality of the two quantities (Eq. 3). In addition, *τ* is inversely proportional to the rate which the network state evolves (Eq. 1a), implying that for networks with large *τ*, the network state evolves much slower compared to networks with small *τ*. This slower evolution of the network’s trajectory results in a reduction of the curvature of the trajectory, which means that the dynamics appear approximately linear at the timescale of the input signal (10–20 timesteps). Furthermore, the approximately linear activity of the neurons, as a result of very large *τ*, can explain the observed drop in dimensionality for *ρ* > 10^4^ in Figs. 4, 5 and 7a. The low estimated dimensionality for these *ρ* values resulted from the fact that the linear activity of neurons in the network could be predicted with a single embedding dimension by the non-parametric estimator, and more than 95% of the variance of the activity could be explained by the first principal component, when using the linear estimator. This explanation can inform us about the computational power of these networks on the input signal; in order for networks to be able to respond to the information provided through the input, their timescale needs to be carefully chosen.

Finally, additional simulations were carried out to check the long-term behaviour of neurons in stable (*g* = 0.9) and unstable (*g* = 1.5) networks for 10^3^ < *ρ* < 10^6^ (results not shown). For these experiments, stimulations were carried out for 3.5 × 10^6^ timesteps, in order to allow the networks’ activities to fully unfold. Two interesting observations were made: firstly, the activities of stable networks approached the origin for *ρ* > 10^5^ in the long run, similar to the diminishing amplitude of oscillation observed in conventional harmonically driven oscillators. Secondly, the activity of networks with *g* = 1.5 was nonlinear at very large timescales ∼10 ^6^, and appeared to span the whole range of activity without any signs of approaching a stable origin, similar to its behaviour in the absence of an input signal. From these observations, we can conclude that at very high *ρ* values, both stable and unstable networks behave in a similar manner as when the input is absent.

## Discussion

### Reservoir Computing Applications

Within the paradigm of reservoir computing, the recurrent network serves as a high-dimensional spatio-temporal kernel acting on an input time series [5]. Its function is to perform a complex nonlinear convolution on the input signal such that, by training linear readout units, the output can be used to approximate the result of a desired computation on the input. This has been shown to have applications, among other areas, in robotic manipulation [27], localisation [4] and navigation [28]. In this latter context, the reservoir acts on an incoming stream of sensory information to either detect events or determine motor actions to complete a task, e.g. navigating between rooms. For such applications the dynamical properties of the reservoir play a crucial role: on the one hand, stability is desirable, in the sense that a similar sensory input should result in similar reservoir activity to guarantee a reliable operation; on the other hand, rich dynamics are also advantageous to ensure that the network activity is expressive enough for the readouts to be appropriately trained.

Our proposed methodology can provide insight into the dynamics of the reservoir driven by the appropriate input, such that the recurrent and external connections can be tuned, and to avoid time-consuming trial and error during training. Using the approach taken here, the reservoir can be initialised with a good estimate of a suitable *g* and *τ*, depending on the nature of the input signal and the desired dynamical complexity. One can then either use PCA or nonlinear embedding to visualise the dynamics and measure their dimensionality from the resulting activity of the network. The effect of the two mentioned parameters on the dynamics can be quickly explored to identify different dynamical regimes and hence guide the parameter search during training.

In a different context, there are settings in which the reservoir is connected to more than one external source, but only one of them is active at any given time. This is the case, for example, in Sussillo’s 3-bit flip gate reservoir, in which three sources can inject the same signal into the reservoir through different sets of input weights [16]. In this scenario, although the driving signals are identical for all sources, the network’s stationary points lie in a different subspace of the state-space as a result of the different input weights. This shows that not only changing the external force itself, but changing its weights to each neuron it is injected to, can modify the dimensionality and structure of the network’s trajectory. In these cases, one can obtain more information about the interplay between the sources’ effect on the network, by considering both the stationary points and the locally linear dynamics under the effect of either each source in isolation, or a combination of multiple sources being active at the same time.

### Biological Significance

Beyond the possible applications of these methods for reservoir computing, the work presented here can also be linked to features of neural dynamics to help advance both modelling and interpretation of brain behaviour [29]. As an example, a recent study by Shenoy and colleagues has shown that the motor cortex is strongly activated long before the onset of any muscle movement [30]. Furthermore, they showed that this pre-movement activity lies in the nullspace of the output connections projecting to the muscles. This means that even though the motor cortex is engaged in complex internal dynamics [31], it has no downstream effect and hence does not initiate any motion. This behaviour allows the motor cortex to integrate information coming from upstream regions of the brain without causing any undesirable, premature movement [10, 32].

The subspace where the network activity lies during preparation without affecting downstream regions could be considered an attractor of specific dimensionality determined by the input from upstream regions. Signals in a brain network such as the motor cortex can be thought of as having two important functions: to transfer information from other brain areas and to affect the dimensionality of the region’s dynamics so that this information is reliably processed and results in a useful motor output. Importantly, if the input signal can force the network into a low-dimensional subspace, then it means that this subspace can become a nullspace by only training the linear output weights to the muscles. This can result in the preparatory activity being performed naturally, without the requirement for a separate gating or threshold mechanism, but rather through the presence of a reliable and reproducible attractor [9, 33]. By extending this idea, one could speculate that other brain regions could use these dynamical properties to establish or interrupt communication, simply through their dynamics. Evidence suggests that such interactions also exist between other parts of the motor cortex [34, 35]; hence the line of enquiry using the methods presented in this study could help uncover some of the unknown mechanisms of cortical communication and information processing.

Interestingly, the nonlinear attractor dimensionality estimator has previously been used by Tajima et al. [17] to show that the attractor complexity of brain dynamics is higher in downstream (cognitive) areas, compared to the upstream (sensory) areas from which they receive direct input. Results have also shown that the attractor complexity in downstream areas of awake monkeys is significantly higher than that of anaesthetised monkeys—which lead to the conclusion that the conscious state and regions associated with higher cognitive processing are characterised by an increased attractor complexity. From our viewpoint in this article, we can hypothesise that the increased dynamic complexity of these cognitively relevant areas could be partially explained by a change in the input signals coming from upstream sensory areas. Furthermore, changes in global power spectrum in the anaesthetised brain [36] could be responsible for maintaining the necessary brain activity without inducing any actions, by restricting dynamics to a particular subspace.

## Conclusion

We have presented a novel method for finding and visualising the stationary point trajectory and locally linear dynamics of externally driven, continuous-time recurrent neural networks operating in different dynamical regimes. Combining this method with standard visualisation techniques we could gain further insight into the dynamics of random networks and examine a resonance-like phenomenon in harmonically driven stable networks.

On a separate line of enquiry, we studied two different methods for estimating the underlying attractor dimensionality—linear estimation via principal component analysis and nonlinear estimation through non-parametric regression and delay embedding [17]. These two methods consistently indicate that (1) unstable networks have more complex and higher-dimensional attractors than stable ones as a result of the appearance of saddle points for smaller values of the driving signal, (2) both stable and unstable networks show a greater complexity at an intermediate value of *ρ*, with a decreasing effect of the input on the network activity for *ρ* larger than these intermediate values, and (3) the results for attractor dimensionality are robust for stable networks (*g* < 1) between 200 and 2000 neurons.

Finally, we used a simple visualisation technique to identify transitions in the geometric form of attractors of dimensionality three in unstable networks (*g* > 1), for small changes of the driving frequency.

## Footnotes

1 We note that *S*(*t*) is not exactly a force, as it is applied to the first-order time derivative of *x*, and not to the second. However, informally the name bears a similar meaning and is a useful analogy.
